# Chitosan/Virgin Coconut Oil-Based Emulsions Doped with Photosensitive Curcumin Loaded Capsules: A Functional Carrier to Topical Treatment

**DOI:** 10.3390/polym16050641

**Published:** 2024-02-27

**Authors:** Luísa C. Rodrigues, Adriana P. Ribeiro, Simone S. Silva, Rui L. Reis

**Affiliations:** 1 3B’s Research Group, I3Bs—Research Institute on Biomaterials, Biodegradables and Biomimetics, University of Minho, Headquarters of the European Institute of Excellence on Tissue Engineering and Regenerative Medicine, AvePark, Parque de Ciência e Tecnologia, Zona Industrial da Gandra, Barco, 4805-017 Guimarães, Portugal; b12081@i3bs.uminho.pt (A.P.R.); rgreis@i3bs.uminho.pt (R.L.R.); 2 ICVS/3B’s—PT Government Associate Laboratory, 4710-057 Braga/Guimarães, Portugal

**Keywords:** functional drug carrier, curcumin, natural polymers, chitosan, emulsion solution, virgin coconut oil

## Abstract

In recent years, there has been a growing interest in developing smart drug delivery systems based on natural resources combined with stimulus-sensitive elements. This trend aims to formulate innovative and sustainable delivery platforms tailored for topical applications. This work proposed the use of layer-by-layer (LbL) methodology to fabricate biocompatible photo-responsive multilayer systems. These systems are composed of a polyoxometalate inorganic salt (POM) ([NaP_5_W_30_O_110_]^14−^) and a natural origin polymer, chitosan (CHT). Curcumin (CUR), a natural bioactive compound, was incorporated to enhance the functionality of these systems during the formation of hollow capsules. The capsules produced, with sizes between 2–5µm (SEM), were further dispersed into CHT/VCO (virgin coconut oil) emulsion solutions that were casted into molds and dried at 37 °C for 48 h. The system presented a higher water uptake in PBS than in acidic conditions, still significantly lower than that earlier reported to other CHT/VCO-based systems. The drug release profile is not significantly influenced by the medium pH reaching a maximum of 37% ± 1% after 48 h. The antioxidant performance of the designed structures was further studied, suggesting a synergistic beneficial effect resulting from CUR, POM, and VCO individual bioactivities. The increased amount of those excipients released to the media over time promoted an increase in the antioxidant activity of the system, reaching a maximum of 38.1% ± 0.1% after 48 h. This work represents a promising step towards developing advanced, sustainable drug delivery systems for topical applications.

## 1. Introduction

Topical drug delivery holds immense promise as an avenue for localized and targeted therapy, offering advantages such as reduced systemic side effects and improved patient adherence [1,2]. Topical product absorption generally occurs in the stratum corneum skin layer, being restricted to molecules relatively smaller in size, with a lipophilic or amphiphilic nature, and nonirritating, which limits the range of deliverable drugs [2,3,4]. To overcome the limitations and drawbacks regarding permeation and bioavailability of drugs, attention has been focused not only on the active ingredient but also on the form and composition of the entire formulation, envisioned to enhance drug permeation and solubility and increase its delivery [2,3,4]. Therefore, the protection and preservation of therapeutic molecules during their release pathway remain required for developing effective drug carrier vehicle applications. The design of carriers able to ensure sustainable drug delivery has been a focus of research as it promotes low toxicity, high stability through cargo protection, and targeting efficiency. Therapeutics delivery using those systems will allow the assessment of different biomedical and pharmaceutical applications, particularly in treating local skin and systemic diseases [5,6,7].

Drug delivery technologies encompass many carriers, including lipid carriers, metal nanoparticles, or polymeric carriers, with their design focused on their properties’ modulation such as hydrophobicity, biodegradability, size, shape, surface charge, and toxicity, ensuring adjusted delivery, biodistribution, cellular uptake, and biocompatibility [8]. The use of liposomes as carriers was reported to increase the effectiveness and reduce the side effects of various active agents [9]. Polyacrylate gel matrix containing a liposomal of *Nigella sativa* seed oil and VCO prolonged drug penetration and increased drug retention in the skin, promoting improved efficiency, suggesting potential to be an effective drug delivery system for dermatological problems [9].

Recently, increased attention has been devoted to developing therapeutic drug carriers able to enhance the effect of the loaded drug, playing an active role in addressing medical challenges [8].

Additionally, incorporating stimulus-sensitive moieties into the carriers leads to the generation of “smart” delivery systems. This innovation allows a spatio-temporal control of the drug release and enhancing local bioavailability and enables therapies with reduced drug doses and low administration frequencies. This approach also aims to increase efficacy while minimizing potential side effects and off-targeting toxicity [10].

In this work, we propose the use of the Layer-by-Layer (LbL) methodology to prepare the carriers. LbL assembly is a simple and highly versatile method to fabricate robust and highly ordered nanostructured coatings over almost any type of substrate [11]. We focus on designing biocompatible photo-responsive multilayer systems using a polyoxometalate inorganic salt (POM) ([NaP_5_W_30_O_110_]^14−^) and a natural origin polymer, chitosan (CHT). CHT is a biocompatible, biodegradable, and FDA-approved biopolymer known in wound management for its hemostatic and anti-inflammatory properties and for being a wound healing accelerator [12,13]. Besides those biological properties, its cationic nature makes it the obvious candidate to integrate the LbL construction with the anionic POM. Moreover, earlier published studies have already shown evidence that POMs exhibit biological activities in vitro and in vivo, such as antitumor, antimicrobial, antioxidant, and antidiabetic [14,15].

This approach offers the possibility of creating materials with a light-stimuli response through the exploitation of the photo-reduction properties of the POM, achieving a spatial controlled disruption of the assembled layers due to the weakening of the electrostatic interactions between the layers, leading to capsule destruction and consequent content release [16].

Incorporating natural phytochemical extracts with dermatological activity, as curcumin (CUR) from the turmeric curcuma longa, allows a more environmentally and economically advantageous approach, as it explores natural resources with high economical potential as health-improving solutions. The natural bioactive compound CUR selection relies on its antioxidant, anti-inflammatory, tissue protective, antibacterial, and wound healing properties [17,18,19]. However, it presents some limitations in its application as a therapeutic agent due to its rapid metabolism and hydrophobic properties, with consequent limited solubility, stability, and bioavailability [19]. Therefore, the design of an approach able to deliver CUR locally to an injury site or for disease treatment applications is of increasing interest [17]. The resource’s incorporation into biomaterials has been a widely explored strategy going from hydrogels to electrospun fibers or nanoparticles, according to the envisioned application [17,19,20,21,22,23].

For wound healing applications, many studies using nanoparticles to deliver CUR have shown significant improvements to the healing process compared to the isolated biomaterial or CUR alone [17,19,20,24]. It was also reported through in vitro studies, the reduced cytotoxic effect of CUR in human skin fibroblast when the CUR was combined within the biomaterial in the form of film, hydrogel, nanoparticles, liposomes, nanofibers, wafers, or sponges [19]. Particularly, earlier studies have reported that curcumin-loaded poly(ε-caprolactone)-poly(ethylene glycol)-poly(ε-caprolactone) nanoparticles presented a reduction of the commonly observed toxic effects of curcumin [19,25,26]. Moreover, its application to a full-thickness wound model in adult female Wistar rats presented, 21 days after application, a wound closure ratio of 93.3 ± 5.6% [26].

**Figure 1:**
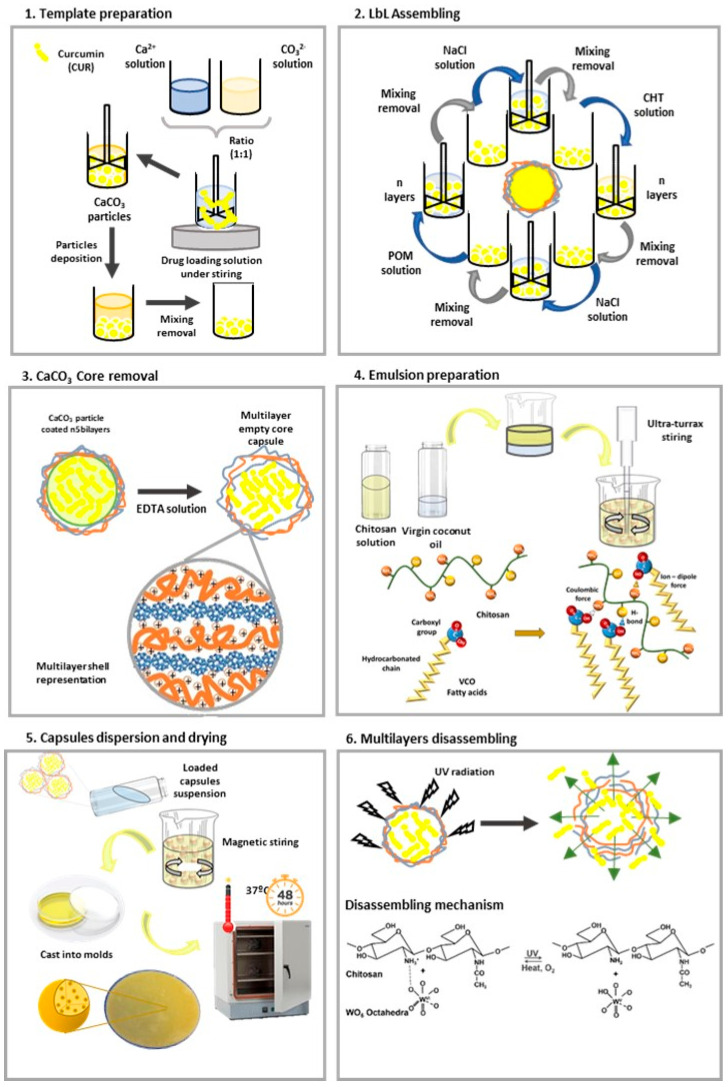
Representation of the methodology employed to prepare the CHT/VCO/ CUR loaded capsules system. Corresponding the Scheme (**1**–**3**) to the CUR loaded capsules preparation; (**4**) to emulsion solution preparation; (**5**) to capsules dispersion and emulsion film molding and drying; and (**6**) to the mechanism of release of CUR from the loaded LbL capsules employed.

The dispersion of the loaded LbL-capsules into emulsion films produced by the association of CHT with virgin coconut oil (VCO) serves as a topical patch to protect wounds, keep the moist environment, and locally deliver the CUR. VCO has been pointed out as a health promoter due to its anti-fungal, antibacterial, anti-inflammatory, anti-aging, and antioxidant properties [27,28]. Further, earlier research has shown that combining VCO with natural polymers, e.g., gellan gum, chitosan, or corn starch [29,30], produces an emulsion-based system that is valuable for many applications. In our previous studies combining VCO with chitosan (CHT), a natural polymer and emulsifier, emulsion films were produced with adequate features to be used as a drug delivery vehicle and a topical patch for wound care [27,28]. In the present study, we hypothesized that dispersed CUR-loaded capsules can be used to design a multifunctional device to be topically applied on the wound site. This work aims to propose a rational design (Figure 1) and characterization of tailored hollow capsules as stimuli-responsive carriers dispersed on emulsion films and their subsequent stepwise screening for physico-chemical properties, delivery efficiency, and bioactivity as a proof-of-concept of the system design and a preliminary evaluation of its behavior, envisioning their use as carrier system for topical application.

## 2. Materials and Methods

### 2.1. Materials

Medium molecular weight chitosan (CHT) (190,000–310,000 Da) from crab shells with ca. 85% deacetylation degree was supplied by Sigma-Aldrich (St. Louis, MO, USA) (practical grade) and used after purification according to a reprecipitation methodology [31].

Polyoxometalate salt ((NH_4_)_14_[NaP_5_W_30_O_110_].31H_2_O) (POM) was prepared according to an earlier reported methodology [32], starting from an heteropolytungstate solution prepared with sodium tungstate dihydrate purum, ≥99% reagent supplied by Sigma-Aldrich (St. Louis, MO, USA).

Curcumin (CUR) powder from Curcuma longa (Turmeric), was used as supplied by Sigma-Aldrich (St. Louis, MO, USA).

Virgin coconut oil (VCO), a commercial product, produced by Copra Indústria Alimentícia Ltda, Maceió, Brasil, was used as received. The composition of the VCO’s main saturated fatty acids and their percentage presence (%) was provided by the manufacturer, as follows: lauric acid, C12–43.5%; myristic acid, C14–18.4%; palmitic acid, C16–10.3%; oleic acid, C18–8.6%; caprylic acid, C8–6.8%; stearic acid, C18–2.7%; capric acid, C10–5.4%; and caproic acid, C2–0.5%.

All the other used reagents and solvents were of analytical grade and used as received.

### 2.2. Methods

#### 2.2.1. Solutions Preparation

CHT polymeric solution was prepared in acetic acid (1%), at a concentration of 1 mg/mL and left to homogenize overnight at room temperature (RT), and its pH was further adjusted to 5.5 with a small addition of NaOH solution (0.1 M).

POM solution was prepared on water with a 0.5 mM concentration at RT and protected from light.

Calcium chloride (CaCl_2_) and sodium carbonate (Na_2_CO_3_) aqueous solutions were prepared at room temperature and a 1 M concentration. Sodium chloride (NaCl) aqueous solution was similarly prepared with a concentration of 0.15 M.

#### 2.2.2. Hollow Capsules Preparation

In a typical coprecipitation reaction, the aqueous solutions of anhydrous sodium carbonate and calcium chloride were used to produce calcium carbonate particles, which were further used as templates [5]. Briefly, 0.1 mg of CUR was added to 1 mL of water to produce the precipitation medium. Each 100 μL of the sodium carbonate solution was added followed by 100 μL of calcium chloride with rapid stirring for 30 s. As a control, empty capsules were fabricated using ultra-pure water as a precipitation medium. The synthesized calcium carbonate (CaCO_3_) particles were further left to react and precipitate for 15 min. Then the supernatant was gently removed and replaced by ultrapure water to remove residual products. This washing procedure was repeated twice.

After washing the calcium carbonate templates, multilayer shells were fabricated using the following sequential steps for five times, ensuring the achievement of the desired number of bilayers: (1) immersion of templates into a 1 mg/mL CHT solution for 10 min under stirring, further on, gently remove the supernatant after particles precipitation; (2) addition of 0.15 M NaCl solution for 5 min under stirring, after each is removed; (3) immersion in 0.5 mM POM solution for 10 min under stirring followed by supernatant removal; and (4) rinse in 0.15 M NaCl solution under stirring for 5 min. The multilayers were assembled to obtain a configuration of up to 5 bilayers. Between each immersion, the system was rinsed in the NaCl solution to ensure the removal of the weakly adsorbed fragments of CHT and POM. After the desired coating conclusion, the beads were immersed in an ethylenediaminetetraacetic acid (EDTA) freshly prepared solution (0.1 M) to chelate its sacrificial core, to achieve hollow capsules with a liquified core with and without CUR.

#### 2.2.3. Curcumin Release from Capsules Conditions Assessment

4 amounts of NaOH 0.1 M were added to 500 µL of capsules dispersed solution (A-50, B-100, C-200, and D-500 µL) to neutralize them, being further washed with osmotized water; also 4 irradiation times frames were tested to study its influence in the release of the capsules content (0, 5, 10 and 15 min).

#### 2.2.4. CHT/VCO-Based Emulsion Preparation

The CHT/VCO-based emulsion was prepared using an earlier reported methodology [27]. Briefly, VCO (1 vol%), and glycerol (1 vol%) were added to 10 mL CHT 1% (*w*/*v*) solution and homogenized at 10,000 rpm for 15 min using a high-speed homogenizer (Ultra Turrax, T18 Basic, IKA-Werke GmbH & Co. KG, Staufen im Breisgau, Germany).

Empty or CUR-loaded capsules were added to the prepared solution at a 10% V7V proportion. The system was homogenized under moderated magnetic stirring for 2 min, and the solutions were casted onto circular petri plates (d = 5.5 cm) and dried at 37 °C. The structures without capsules were prepared using a similar procedure, still without the capsule’s addition step. The sample prepared will be described according to the nomenclature described in Table 1.

#### 2.2.5. Physico-Chemical Characterization

##### Structural Features (QCM-D)

CHT and POM ability to interact with each other in a liquid environment was evaluated using 2D planar gold surfaces, being monitored by a quartz-crystal microbalance with dissipation monitoring (QCM-D, QSense E4, Västra Frölunda, Sweden). The measuring was performed according to an earlier described procedure, using freshly prepared solutions [16]. Briefly, 1 mg/mL CHT, 0.5 mM POM, and 0.15 M NaCl solutions were alternatively introduced into the measuring chamber at a flow rate of 50 μL·min^−1^, and the frequency (ΔF) and in dissipation (ΔD) variations were monitored at real-time. Voigt viscoelastic model was implemented in the QTools software (version: 3.1.25.604) from QSense, to obtain the thickness of the sequentially adsorbed layers.

##### Thermal Analysis–Differential Scanning Calorimetry (DSC)

Sections from the prepared films weighing 3–5 mg were presented for DSC analysis in 40 μL aluminum pans covered with a suitable aluminum cover. DSC analysis was performed between 25 and 300 °C at a heating rate of 5 °C min^−1^ under a flowing nitrogen atmosphere in a DSC Q100 instrument from T. A. INSTRUMENTS. An indium standard was used to make temperature and enthalpy calibration.

##### Morphology Assessment (SEM)

The morphology of the film samples was observed using a JEOL analytical scanning electron microscope (SEM), JSM-6010LV model, equipped with an energy dispersive spectroscope (EDS). Before SEM analysis, samples were freeze-dried and further coated with gold using a Quorum/Polaron model E 6700 equipment. The SEM analysis was performed with an acceleration voltage of 5.00 kV and magnification of 5000× for capsules and 1000× and 5000× for films.

##### Swelling and Release Assays

1 cm^2^ sections of the membrane were soaked into sodium phosphate dibasic (pH = 7) or citric acid-phosphate (pH = 5) buffer solutions at 37 °C to access the water uptake and CUR release profile of the proposed system.

**Water uptake:** The swollen samples were extracted from each buffer solution at predefined time points (15 min, 30 min, 45 min, 1 h, 2 h, 3 h, 4 h, 5 h, 6 h, 24 h, 2 days, and 4 days), and the excess of solution removed with filter paper (Filter Lab, Spain). Thus, the weight of the samples was accessed using an analytical balance (Denver Instrument, Germany). All the assays were performed in triplicate (*n* = 3). The water uptake was calculated as follows:

(1) $$Water\;uptake\;\left(\%\right)=\frac{W_{s}-W_{d}}{W_{d}}\times 100\;\;\;\;\;\;\;$$

where *W_s_* and *W_d_* are the weights of swollen and dried films, respectively.

**CUR release:** Similarly to the water uptake procedure earlier described, the samples were soaked into sodium phosphate dibasic (pH = 7.4) or citric acid-phosphate (pH = 5) buffer solutions at 37 °C to access the Curcumin release profile of the system for 48 h, under mild agitation to improve the diffusion through the incubating media. At predefined time points, the surrounding buffer media were removed from each container, and the experiments were performed in triplicate (*n* = 3). The surrounding buffer media volume was maintained constant throughout the experiment. The emulsion with dispersed capsules was tested before and after 10 min irradiation to ensure the release of the capsule content and the possible influence of the capsule degradation residues in the media.

A calibration curve was constructed based on ten CUR concentrations, between 1.0 × 10^−5^ g.mL^−1^ and 1.0 × 10^−4^ g mL^−1^, from which absorbance was measured at 425 nm. The absorbance of the retrieved samples was measured at the same wavelength, and the achieved values were comparatively studied within a previously constructed calibration curve.

##### Antioxidant Activity Assay

The 2,2-diphenyl-1 picrylhydrazyl (DPPH) radical scavenging assay was used to assess the antioxidant activity of the designed system. This simple methodology relies on the color change of the DPPH solution from purple to yellow, which is related to the radical quenching by the antioxidant [33]. The experimental procedure was performed according to earlier reported instructions. Briefly, a volume of 1 mM DPPH solution in ethanol was diluted in 50 mL of ethanol, and further added to 1 cm^2^ sections of the membranes formulations and kept in the dark, at room temperature. The reaction was monitored at pre-established time points (1 h, 3 h, 24 h, and 48 h) by visible absorbance spectroscopy at 517 nm. A blank control, only DPPH diluted solution, was prepared and maintained at the same conditions.

The DPPH radical scavenging activity, AA(%), was determined according to the equation:

(2) $$\mathrm{AA}\left(\%\right)=\frac{A_{0}-A_{1}}{A_{0}}\times 100$$

where A_0_ and A_1_ are, respectively, the absorbances of the control and the samples.

#### 2.2.6. Statistical Data Treatment

The experimental tests were conducted at least in triplicate (*n* = 3), and the achieved data points are presented as the mean standard deviation. GraphPad Prism 5.0 software was used for statistical analysis.

The data that demonstrated a normal distribution were analyzed while using an ordinary two-way ANOVA. In addition, the statistical analysis (*n* = 6) was performed using the Tukey’s multiple comparisons test to determine statistical differences. The significance level between the groups was set for * *p* < 0.05, ** *p* < 0.01, and *** *p* < 0.001. The data were presented as mean ± standard deviation (SD).

## 3. Results and Discussion

**Figure 2:**
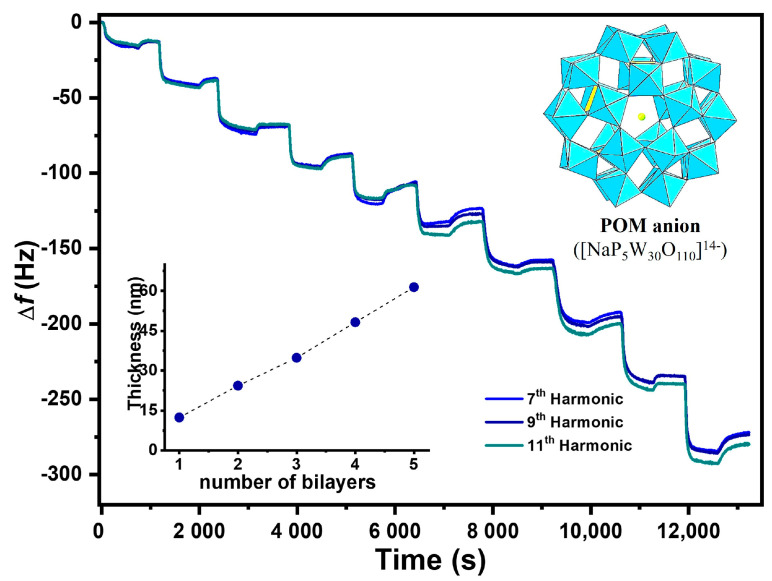
Frequency measurements during LbL assembly were obtained by QCM-D analysis of the (CHT/POM)_5_ 2D system assembly (from the selected 7th, 9th, and 11th harmonics). Bottom inset: Thickness increases as a function of the bilayer number, obtained using a Voigt viscoelastic model. Top insert: POM anion ([NaP_5_W_30_O_110_]^14−^) structure representation.

The resource to drug-loaded polymeric carriers assumed significant interest as a topical application solution particularly devoted to treating skin diseases [34]. The integration of stimuli-response potential to the system allowed a temporal control of the drug release, enhancing its topical bioavailability and reducing the amount of applied drug and the frequency of its administration [10,19,34,35,36]. The resource to the Layer-by-layer method is relevant, considering that it is a simple and highly versatile method to fabricate robust and highly-ordered nanostructured coatings [7,11,37]. Moreover, it allowed the design a biocompatible photo-responsive multilayer system based on a polyoxometalate inorganic salt (POM) ([NaP_5_W_30_O_110_]^14−^) and a natural origin polymer, chitosan. CHT and POM ability to adsorb in a sequential fashion was accessed by QCM-D, using a 2D apparatus. Similarly to earlier reported data [16], a decrease in the frequency was observed for each layer deposition in the 5 multilayers system construction, Figure 2. The estimated thickness also followed a linear increase tendency along the building-up process.

According to this, it was hypothesized that the translation to a 3D system would allow the successful construction of a hollow carrier with light-stimuli response-ability.

The thermal analysis of the 2D-produced multilayer film showed no significant thermal changes promoted by the physico-chemical interaction between polymer and salt on the multilayer construct, Figure S1. A predominance of the thermal profile of the polymer is observed in the multilayer film, with no fusion phenomena being detected in the studied temperature range. The endothermic peak present between below 90 °C is characteristic of CHT and is commonly assigned to the loss of water associated with the hydrophilic groups of CHT [38]. The appearance of this peak suggests that the CHT present in the films was not completely dried, and some bounded water molecules were still not removed during the drying. Also, an exothermic peak starts to appear at the thermogram for T > 280 °C, which is assigned to the thermal degradation of chitosan [38].

**Figure 3:**
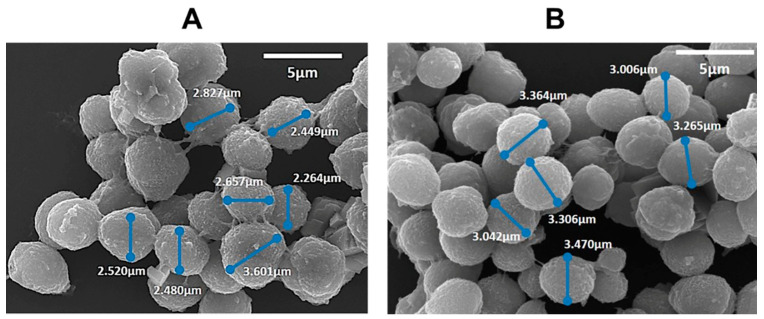
SEM of the (CHT/POM)_5_ non-loaded (**A**) and CUR loaded (**B**) capsules. Scale bar: 5 µm.

The translation from a 2D multilayer film to a 3D system was build-up through an LbL deposition over calcium carbonate particles as sacrificial templates, prepared by co-precipitation of Na_2_CO_3_ and CaCl_2_ solutions under vigorous stirring in a CUR medium. 5 multilayers of CHT and POM were successfully built on the top of the almost spherical CaCO_3_ template. After construction, the core was chelated with EDTA to form hollow microcapsules with encapsulated CUR solution, as a model delivery excipient. The morphology of the designed loaded (CHT/POM)_5_ capsules was evaluated by SEM, Figure 3. The system presents an almost spherical structure similar to the used template and a surficial roughness, which is in accordance with earlier reported morphological observations for capsules prepared through the same methodology [7]. Some aggregation was also observed, which may be ascribed to the air-drying required for SEM samples analysis, which promotes a reduction of the electrostatic repulsion between the multilayers coated particles [39], still due to the reduced coalescence observed is expected a reduced particle aggregation at aqueous medium. Additionally, it was observed an increase in the diameter of the developed capsules when CUR was loaded on the structure, varying from an average of 2.7 ± 0.3 µm for the empty structures to 3.2 ± 0.1 µm for the CUR-loaded ones. This may be related to the positive charge surface of the loaded CUR [40] that may repulse CHT cationic layers, leading to a slight increase of the achieved capsule size [37].

Drug release from the designed capsules was achieved through the photo-reduction properties of the POM salt layers. This allowed a spatial controlled disruption of the assembled layers due to the weakening of the electrostatic interactions between the layers, promoting capsule destruction and consequent content release.

To access the release ability of the (CHT/POM)_5_ capsules loaded with CUR, different conditions were tested to promote the drug release. According to the results, the condition in which the irradiation was preceded by capsules washing with 100 µL of NaOH solution, was considered the better condition for releasing the capsules’ content, and the one selected for further assays. This fact may be due to a stabilization of the charge of the multilayers that may be affected by the CaCO_3_ chelation step in highly acidic media, which is neutralized through this washing step. According to earlier studies, the charge density of CHT is much higher at lower pH, which leads to a compact structure and flat conformation, which may make difficult layers to disassemble [37]. Furthermore, and as expected, an increase of the time frame of the UV light exposure led to an increase of the released content in any of the studied conditions until 10 min of exposure, after which a plateau was reached. According to this, for further release assays, the time of irradiation selected was 10 min.

CHT/VCO-based emulsion systems were included in the system to act as physical support for capsule dispersion. This approach will enhance the system’s potential for further topical application on the damaged skin surface, playing an active role in the healing process due to its intrinsic properties. As previously studied, CHT/VCO-based emulsions are viscous solutions with a non-Newtonian behavior [27], supporting the dispersion of CUR-loaded capsules, without compromising its film-forming ability. After casting into molds, the solutions were dried at 37 °C for 48 h, generating emulsion films with 0.30 ± 0.02 mm of thickness with good stability, handling ability, and flexibility. SEM images of the non-loaded and capsule-loaded films are presented in Figure 3 of the supplementary information. Figure S3B, suggests that the capsules incorporated into the film can cluster together with the oil droplets, making it difficult to see their distribution within the film. The observed droplets at CHT/VCO films surface are similar to earlier reported data [27], where the oil droplets at the films surface present a high heterogeneity of sizes, with a mean value of 10.8 ± 4.6 μm, which are in accordance with the presented data. Moreover, it is important to note that a notable increase in the film’s surface roughness was observed for the capsules loaded films compared to the non-loaded ones (Figure S3A), which may suggest the confirmation of the capsule’s presence within the film structure.

**Figure 4:**
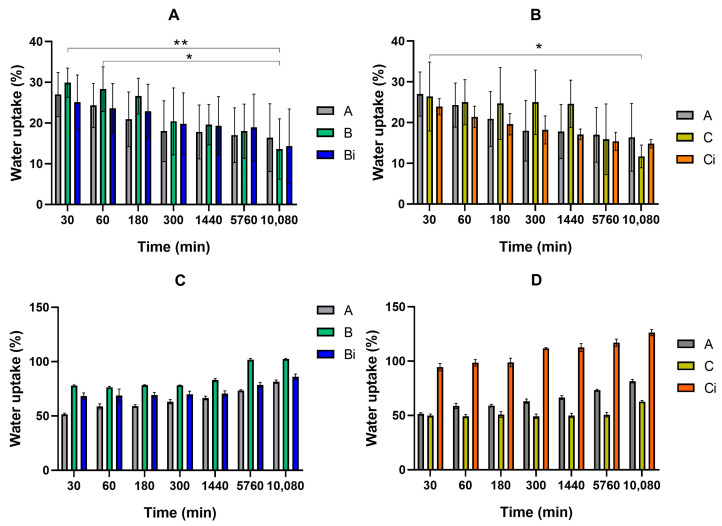
Swelling performance of CHT/VCO emulsion films loaded with CHT/POM capsules with and without curcumin in different pH media, pH 5 buffer (**A**,**B**) and PBS (**C**,**D**), before and after irradiation. Significant differences were assigned with ** and * respectively for *p* < 0.01, and *p* < 0.05.

CHT/VCO-based films with and without dispersed CUR(CHT/POM)_5_ hollow capsules were punched into 6 mm diameter circles and immersed in PBS or pH 5 buffer solutions to evaluate their swelling ability and CUR release profile. The system presents a significantly higher water uptake in PBS than in acid conditions, still not significantly influencing the drug release profile observed in the different media (Figure 4). Almost no statistical differences were found for the degree of swelling at pH 5, while in PBS (pH 7.4) statistical differences were identified between some formulations and the different immersion time points (Figure 4). Furthermore, the presented swelling behavior is significantly less pronounced than what was earlier reported for CHT/VCO-based films [27]. The superabsorbent nature earlier reported was, in this work, controlled due to the presence of 1% glycerol in the emulsion system, which can potentiate the establishment of inter- and intramolecular glucosamine interaction into the emulsion solution, as already has been reported for other CHT-based systems [41,42].

In accordance with the swelling pathway is the observed mass loss tendency. The samples mass loss after drying is more pronounced for the samples in PBS (Table 2), suggesting that a higher water permeation in the system may reduce the interaction between the polymer and the oil fatty acids chains, positively influencing oil migration to the media justifying the increase of the mass loss over time.

**Figure 5:**
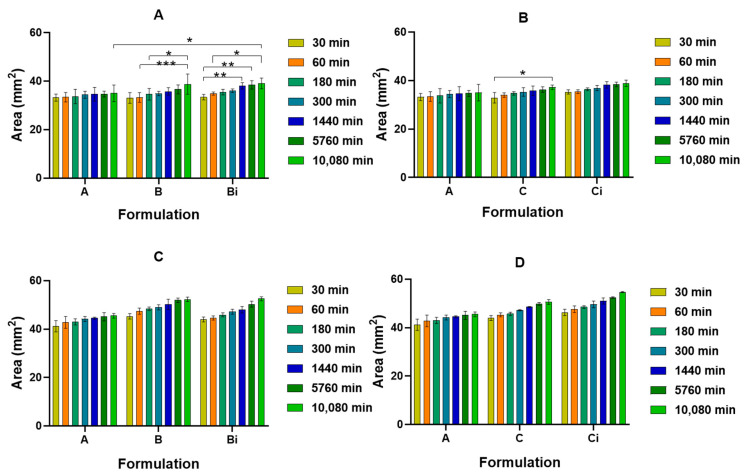
Area variation of CHT/VCO-based emulsion film samples loaded with CHT/POM capsules with and without curcumin in different pH media, pH 5 buffer (**A**,**B**), and PBS (**C**,**D**). Significant differences were assigned with ***, ** and * respectively for *p* < 0.001, *p* < 0.01 and *p* < 0.05.

The area variation studies included in Figure 5, are also in accordance with the observed swelling behavior, being the higher mass increase observed for the samples immersed in PBS, with significantly relevant statistical differences observed (Figure 5C,D). At pH 5 buffer, besides the lower increase observed for the samples in the different compositions, it is also noted that a plateau is achieved in the first minutes of immersion, the differences along time being almost negligible (Figure 5A,B). Particularly regarding the C and Ci formulations, where CUR was present, just for the non-irradiated sample (C) a difference was observed between after 0.5 h and 7 days of immersion in pH 5 buffer.

In PBS, the behavior of the samples is different being considerably influenced by the immersion time and the presence of the CHT/POM hollow capsules, with reportable statistical differences (Figure 5C,D).

**Figure 6:**
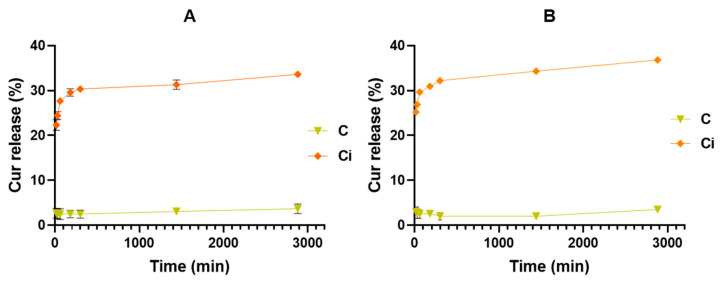
Curcumin release evaluation from CHT/VCO emulsion films loaded with CUR(CHT/POM)_5_ before and after 10 min radiation exposure in different media: (**A**) pH 5 buffer solution and (**B**) PBS.

The release assay was performed at 37 °C and two different pHs. Those conditions were defined according to the application envisioned for the system. According to literature, wound pH may range from 5–9 [43], so a more acid pH (pH = 5) was studied to observe the influence of a slightly acid media on a material based on CHT, a cationic polymer soluble in acid media. The PBS media (pH = 7.4) was employed as it is the most commonly used buffer to study the release of compounds with application in biological systems. According to the data presented in Figure 6, the loaded CUR was successfully released from the films at PBS and pH 5 buffer media. When immersed in the release media, the system becomes hydrated as the water molecules are able to permeate the film structure, which may reduce the interaction between the polymer and the oil fatty acids chains, positively influencing oil migration to the media [27]. According to this, the oil acted as a vehicle to transport the released CUR to the immersion medium. The capsules are not expected to exit the film structure and permeate the skin. We proposed that the loaded capsules dispersed in the film may be disrupted under light stimulation, being the drug released and was able to migrate from the structure. As CUR is a lipophilic compound, its migration may be favored by the emulsion lipophilic nature, being the oil migration mechanism used to transfer it to the release medium [28]. According to this, and as the amount of oil in the emulsion film is higher at the beginning of the assay, it favors the CUR exit from the system to the media, justifying the observed initial burst release of CUR, after being released from the dispersed capsules. A similar behavior has already been reported for other oil-based systems loaded with different drug carriers [28]. Envisioning the intent of developing a topical application, this approach will benefit from the oil ability of promote skin permeability of active agents. Earlier studies have reported the active role of free fatty acids on drugs skin permeation, suggesting that vegetable oils, such as virgin coconut oil, may be successfully used as permeation enhancers with “safety profiles” [44,45]. These oils contain fatty acids able to promote skin permeation through lipid fluidization within the stratum corneum; and might, therefore be able to effectively enhance transdermal drug delivery [44,45].

The release kinetics were markedly influenced by radiation exposure, with low quantities of released CUR being detected from microcapsules not subjected to any irradiation. The reduced amounts detected were almost constant over time and similar for both media 2.5 ± 1.0%, suggesting that it may be due to a residual amount of disrupted capsules dispersed in the system. Considering the irradiated ones, a fast release for the first 2 h is observed, after which the release speed significantly decreases, reaching an almost constant value after 5 h of immersion in both media. Still, a slightly higher amount of CUR was released in PBS than in pH 5 media, respectively 37 ± 1% and 33 ± 1% after 48 h. This trend suggests that the media pH does not significantly influence the release efficiency of CUR, but still overcomes the earlier reported drawback of CUR release from CHT-based systems due to the formation of polyphenol-CHT complexes that retard the effective CUR release [46].

### Antioxidant Activity

**Figure 7:**
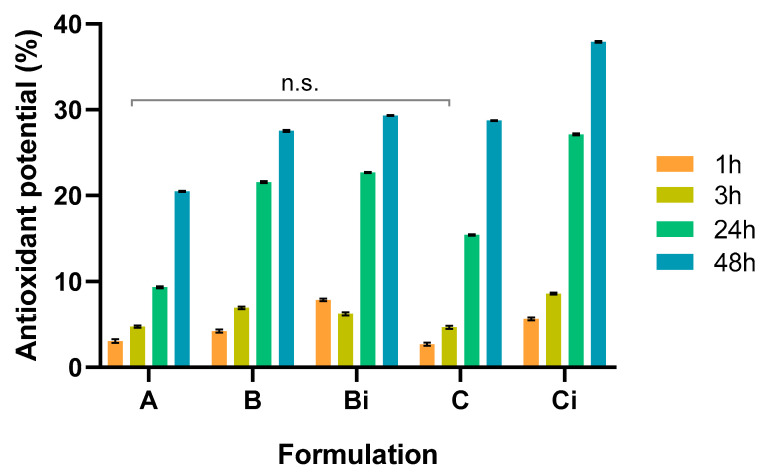
Antioxidant activity evaluation of CHT/VCO emulsion films loaded with CHT/POM capsules with and without curcumin, before and after radiation trigger. Absence of significant differences were assigned with n.s. (mean no significant differences between results).

Damage to DNA, proteins, and lipids is due to ROS generated under oxidative stress conditions. So, it is important to suppress ROS accumulation and augment antioxidant levels. The antioxidant potential of the system was screened through a DPPH assay (Figure 7). All the studied compositions present antioxidant activity, attributed to the presence of VCO in the film, POM at capsule composition, and CUR, the releasable drug [15,19,29,46,47]. Their synergistic effect increased the observed effect for the CHT/VCO film with CUR-loaded capsules after irradiation, which promoted the CUR release to the media. Still, it was possible to observe an increase of the antioxidant potential over time for all the formulations, with significant statistical differences, confirming the influence of the individual antioxidant activity of each intervenient, particularly by VCO, which is released without any stimuli requirement. Furthermore, an increase in the antioxidant activity for the empty irradiated capsules is observed.

This suggests that the disassembly of the capsule layers allows an increase of POM in the media, which is responsible for the enhancement of the antioxidant activity of the system. The results achieved for the CUR-loaded capsules after irradiation are in accordance with the release profile achieved for the system, where the increase of CUR amount released to the media over time promoted an increase in the antioxidant activity of the system [17,19,20,24]. According to this, the proposed design antioxidant performance benefits from the synergistic contribution of VCO, POM, and CUR, being the formulation with higher antioxidant potential the one containing the disrupted CUR loaded capsules.

## 4. Conclusions

Using a multilayer approach, we have successfully conceptualized and developed photo-responsive microcapsules by taking advantage of the self-assembly properties of CHT and POM. Our results demonstrate the feasibility of this technology translation from a 2D to 3D architecture, allowing the construction of micrometric devices using a nonconventional combination of natural resources. The hollow capsules loaded with CUR were further successfully incorporated into a VCO-based emulsion film, forming a multifunctional deliver device.

The permeability of CUR to the exterior was easily tuned by adjusting the radiation exposure. Thus, the multilayer shell proved to be an effective barrier, with the release being activated through irradiation, facilitating sustained release of the microcapsule content. The oil migration from the emulsion film positively influenced CUR released to the immersion medium, being the oil considered a transport vehicle of CUR due to its lipophilic nature.

The observed antioxidant activity for the proposed system resulted from a synergistic effect involving three key components—VCO, POM, and CUR. Remarkably, the system is able to release its content, being the formulation with the disrupted carriers is the one presenting the highest antioxidant potential.

The current findings suggest that the photo-responsive microcapsules (CHT/POM) loaded in VCO-based emulsion films hold promise as drug carriers with intrinsic bioactive potential. Based on this, we are committed to conducting more extensive evaluations in a future work, including the evaluation of the system performance in vitro, ex-vivo, and in vivo to access the permeation of the drug and its effectiveness as a transdermal drug delivery system.

## Figures and Tables

**Table 1:** Samples abbreviation description.

Abbreviation	Sample Composition
A	CHT/VCO
B	CHT/VCO/empty caps
Bi	CHT/VCO/empty caps after irradiation
C	CHT/VCO/curcumin loaded caps
Ci	CHT/VCO/curcumin loaded caps after irradiation

**Table 2:** Mass loss after 7 days in different media.

Composition	Mass Loss (%)		Thickness Variation (%)	
	PBS	pH 5 Buffer	PBS	pH 5 Buffer
A	(37.4 ± 4.2)%	(19.9 ± 6.8)%	(54.8 ± 8.6)%	(15.1 ± 5.0)%
B	(37.4 ± 1.4)%	(18.2 ± 2.7)%	(54.8 ± 9.9)%	(28.6 ± 5.9)%
Bi	(39.7 ± 5.5)%	(18.9 ± 6.1)%	(86.9 ± 8.8)%	(35.6 ± 8.8)%
C	(39.9 ± 2.3)%	(39.5 ± 4.7)%	(63.1 ± 4.4)%	(25.3 ± 3.6)%
Ci	(53.1 ± 2.6)%	(62.5 ± 5.9)%	(131.0 ± 5.1)%	(68.1 ± 6.5)%

## Data Availability

The raw data supporting the conclusions of this article will be made available by the authors on request.

## Supplementary Materials

The following supporting information can be downloaded at: https://www.mdpi.com/article/10.3390/polym16050641/s1, Figure S1: DSC thermogram of the polymeric matrix, inorganic salt, and (CHT/POM)_5_ film; Figure S2: Curcumin release from (CHT/POM)_5_ microcapsules subjected to different irradiation times and release conditions (A-50 µL, B-100 µL, C-200 µL and D-500 µL addition of 0.1 M NaOH solution); Figure S3: SEM micrographs of CHT/VCO films (A) non-loaded and (B) loaded with CUR(CHT/POM)5.
