# Supplementary material for: Chitosan/Virgin Coconut Oil-Based Emulsions Doped with Photosensitive Curcumin Loaded Capsules: A Functional Carrier to Topical Treatment
Source: Polymers (Basel). 2024 Feb 27;16(5):641. doi: 10.3390/polym16050641 (PMC10935360; doi:10.3390/polym16050641)
Supplement: Supplementary file 1 [file polymers-16-00641-s001.zip › polymers-2800530-supplementary.pdf]

Supplementary information

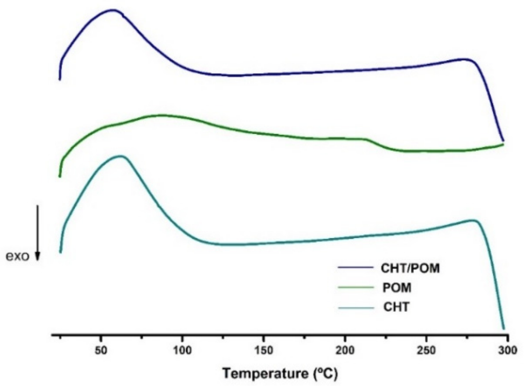

Figure S1. DSC thermogram of the polymeric matrix, inorganic salt, and $(CHT/POM)_5$ film.

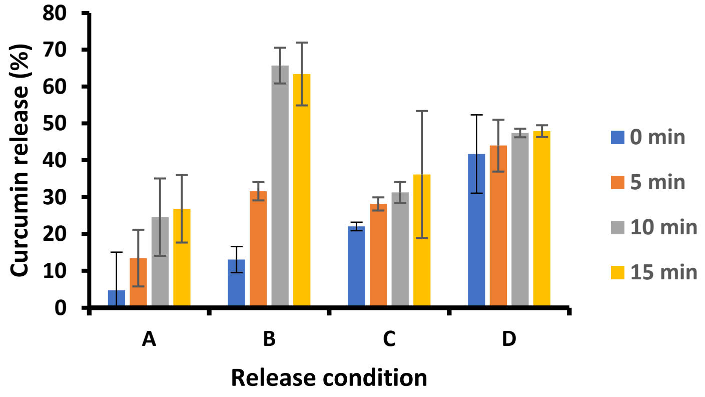

Figure S2. Curcumin release from (CHT/POM)5 microcapsules subjected to different irradiation times and release conditions (A-50 μL, B-100 μL, C-200 μL and D-500 μL addition of 0.1M NaOH solution)

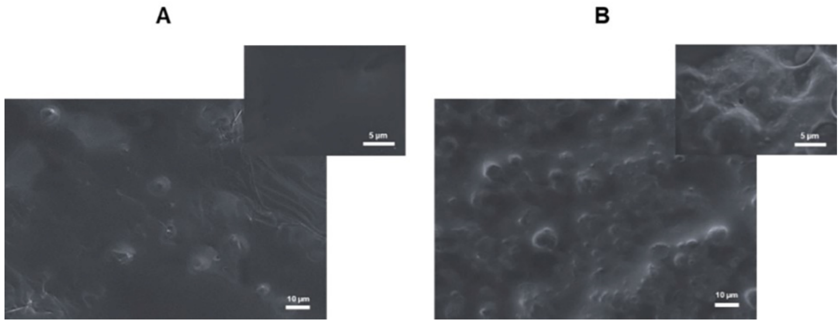

Figure S3. SEM micrographs of CHT/VCO films (A) non-loaded and (B) loaded with CUR(CHT/POM)5.
